# Expression Analysis of the CRISP2, CATSPER1, PATE1 and SEMG1 in the Sperm of Men with Idiopathic Asthenozoospermia

**Published:** 2019

**Authors:** Zohreh Heidary, Majid Zaki-Dizaji, Kioomars Saliminejad, Hamid Reza Khorramkhorshid

**Affiliations:** 1- Reproductive Biotechnology Research Center, Avicenna Research Institute, ACECR, Tehran, Iran; 2- Genetics Research Center, University of Social Welfare and Rehabilitation Sciences, Tehran, Iran

**Keywords:** Asthenozoospermia, CATSPER1, CRISP2, Gene expression, Male infertility, PATE1, Real-time PCR, SEMG1, Sperm motility

## Abstract

**Background:**

The purpose of this study was to analyze the expression level of CRISP2, CATSPER1, PATE1 and SEMG1 genes in the sperm of men with asthenozoospermia (AZS). AZS is a cause of infertility in men in which the motility of the sperm is reduced. So far, a few genes have been associated with AZS; however, in most of the cases, its molecular etiology is unclear.

**Methods:**

A total of 35 subjects with idiopathic AZS and 35 fertile and healthy men as control were included. In study after total RNA extraction and cDNA synthesis, relative quantification was performed. B2M was used as the normalizer gene and fold change was calculated by 2^−ΔΔCt^ method. Mann-Whitney test was used to compare the expression levels between the case and control groups with significance level of p<0.05.

**Results:**

Our results showed that CRISP2 (p=0.03) and SEMG1 (p=0.03) were significantly down-and up-regulated in AZS men respectively compared to the controls. But CATSPER1 and PATE1 did not show significant changes.

**Conclusion:**

Down-regulation of CRISP2 and up-regulation of SEMG1 were associated with AZS, which could be suggested as the potential candidate genes for the development of a diagnostic marker or potentially for more studies for treatment of AZS.

## Introduction

Infertility of men accounts for ∼50% of all infertile cases, and genetic causes are responsible for 15–30% of all male infertility (1). Asthenozoospermia (AZS) as a cause of infertility in men is defined by absent or decreasing forward sperm motility (Progressive motility <32%) (2–8). AZS could be seen as a pure isolated condition or could be coupled with additional sperm abnormalities. Isolated form of AZS is considered as one of the causes of infertility in men, approximately accounts for 20% of infertile men and in more than 60% of cases this condition is associated with decreased number of sperm (Oligoasthenozoospermia) and/or abnormal sperm morphologies (Oligoasthenoterato-and asthenoteratozoospermia) (9, 10).

A few studies have been reported to find the etiology of AZS (11). Differentially expressed genes including CATSPER1 (Cation Channel Sperm Associated 1) in mouse sperm (12), and PATE1 (Prostate and Testis Expressed 1), SEMG1 (Semenogelin 1), and CRISP2 (Cysteine-Rich Secretory Protein 2), in the human study are apparently related to AZS (13–15). CRISP2 is the only member of CRISP family which is expressed in the testis (16, 17) in an androgen-independent manner (18). CRISP2 protein is located in the acrosome and the outer dense fibers of the sperm tail (19–23). This protein may be secreted from the acrosome during the acrosome reaction (24) or be implicated in sperm-egg fusion (26) and it may modulate sperm flagellar motility (25). CATSPER1 is a voltage-gated Ca^2+^-permeable channel specifically expressed in the sperm flagellum especially on the plasma membrane of sperm tail (27). In a murine model of AZS, up-regulation of CATSPER1 increased the sperm intracellular Ca^2+^ concentration, sperm concentration, and percentages of sperm activity and overall sperm motility (12). It seems that CATSPER1is essential for sperm motility and hyperactivation through regulation of calcium concentration (28). PATE1 was associated with the age-related functions of testis and epididymis. Comparison of aged men and young asthenozoospermic men showed a similar expression level of PATE1. Previous studies on antibody blocking of PATE1 showed that it involved in sperm motility and sperm-egg penetration (14). SEMG1 as another candidate gene for AZS is expressed in seminal vesicles (29, 30) and has been associated with the spermatogenesis process (31). In a study by Yu et al. (2014), SEMG1 was six-fold up-regulated in AZS in comparison to fertile men (15). It appears that SEMG1 as a major structural protein of semen coagulum is involved in the semen coagulation and spermatozoa immobilization to inhibit human sperm capacitation (32). By a literature review of the previous studies in the sperm of men with AZS and healthy individuals, four candidate genes including CRISP2, CATSPER1, PATE1 and SEMG1 were selected to evaluate the expression levels of these genes in our population.

## Methods

### Subjects:

A total of 35 subjects with idiopathic AZS were included. The semen samples were collected between May 2017 and October 2017 from Avicenna Fertility Center, Tehran, Iran. Infertile men with alcohol, drug, tobacco and substance abuse were also excluded. The subjects with known diseases such as cryptorchidism, varicocele, orchitis, epididymitis, endocrine hypogonadism, obstruction of the vas deferens, microdeletions of the Y chromosome and karyotype anomalies were excluded from the study as well. The control group involved 35 age-matched fertile healthy men whose semen analysis showed normal results based on WHO criteria, and had fathered at least one child. The study was approved by the Ethical Committee of the Avicenna Research Institute. All subjects were informed about the purpose of sample collection and informed written consents were obtained.

The semen samples were obtained by masturbation after 3–7 days of sexual abstinence. Semen analyses were performed according to the World Health Organization (WHO) recommendations (2). The inclusion criteria were asthenozoospermic men with the concentration of >20×10^6^ *sperm/ml* in the asthenozoospermic group and rapid forward progressive motile sperm (Grade A) of <25%, and total progressive motile sperm (Grade A+B) of <50% (2).

### Total RNA extraction and cDNA synthesis:

Total RNA was extracted from sperm pellets using RN easy mini kit (Qiagen, Germany) following the manufacturer’s instructions. The quality and concentration of total RNA were determined by spectrophotometry using Nanodrop 2000 (Thermo Scientific, USA). The extracted RNA samples were stored at −80°*C*. The cDNA was synthesized from 0.5 *μg* of total RNA using PrimeScript RT reagent kit according to the manufacturer’s instructions (TakaraBIO, Shiga, Japan).

### Quantitative real-time PCR:

Real-time PCR was performed using SYBR Premix DimerEraser kit gene according to the manufacturer’s instructions (Takara, Shiga, Japan). All the reactions were carried out on a Rotor-Gene Q real-time PCR instrument (Qiagen Inc., Germany) according to the manufacturers‘ instructions. Briefly, 5 *μl* of SYBR Premix, 5 *pmol* of each primer and 50 *ng* of cDNA as template were used in a final volume of 10 *μl*. The amplification reactions were thermally cycled as follows: denaturation at 95°*C* for 30 *s*, followed by 40 cycles of denaturation at 95°*C* for 30 *s*, annealing at 60°*C* for 10 *s*, and extension at 72°*C* for 15 *s*. Human beta-2-microglobulin (B2M) (33) was selected as a normalization standard and fold change in expression of each target mRNA relative to B2M was calculated based on 2^−ΔΔCt^ relative expression formula. The primer sets were designed in exon-junction or between two adjacent exons separated by a large intron to ensure the amplification of RNA and not genomic DNA. The primers used for detecting the expression of genes are listed in table 1.

**Table 1.** Primer sequences and their related PCR product sizes used for real-time RT-PCR

**Gene**	**Forward primer (5′--->3′)**	**Reverse primer(5′--->3′)**	**Size (*bp*)**
**CRISP2**	TGCCATTATTGTCCTGCTGGT	CATGTTCACAGCCAGTTGTATTCT	187
**CATSPER1**	AAGGGCAATTTCAGAAACGCA	TCAAAGGCCAAGGATTGGGTTA	157
**PATE1**	TCTGCTGCTTTAGGGCGTTAT	GGTGGCACATCCTACACTGA	120
**SEMG1**	CCAGACACCAACATGGATCTCA	TGAGGTCAACTGACACCTTGATA	179
**B2M**	CGAGATGTCTTGCTCCGTG	TCCATTCTCTGCTGGATGAGG	118

### Statistical analysis:

Data are expressed as mean± standard deviation and median, 10–90 percentiles. Shapiro-Wilk normality test was used to analyze the distribution of the values for each variable in each group.

The differences of mRNAs expression in the ejaculated sperm between astheno-and normozoospermic men were determined by a Mann-Whitney test. The p<0.05 were considered significant. To assess relevant sensitivity and specificity of expression assay, the receiver Operating characteristic (ROC) curve analysis, and area under the curve (AUC) were used. The analyses were performed by SPSS software version 23.0 (Chicago, IL, USA).

## Results

Descriptive analysis of samples showed that the mean ages in the AZS and control groups were 37±6 and 35±4 years, respectively. The mean sperm count in the AZS men and control group were 33.8±12 and 43.2±16 million *sperm/ml*, respectively.

The mRNA expression levels of CRISP2, CATSPER1, PATE1, and SEMG1 gene were examined by qRT-PCR in the ejaculated sperm samples from the AZS and control groups. Our results showed that no significant differences in CATSPER1, and PATE1 mRNA expression were found between the two groups. On the other hand, CRISP2 (p=0.03) and SEMG1 (p=0.03) were significantly down-and up-regulated respectively in the semen samples from asthenozoospermic men compared to normozoospermic controls (Table 2).

**Table 2.** Expression levels (Fold change) of CATSPER1, PATE1, CRISP2, and SEMG1 mRNAs in sperm of asthenozoospermia men and control groups

**Genes**	**p-value**	**Patients Mean±SD**	**Controls Mean±SD**
**SEMG1**	0.03	6.89±8.53	4.00±5.77
**CATSPER1**	0.58	1.88±2.52	2.24±2.43
**CRISP2**	0.03	3.72±2.01	4.81±2.24
**PATE1**	0.67	2.65±2.99	1.96±2.06

**Figure 1.**
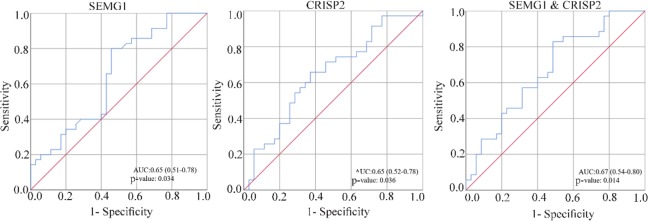
ROC curve analysis of expression data. ROC curve comparing sensitivity and specificity of expression data for detection of AZS patients. ROC curve analyses suggest that expression of SEMG1 and CRISP2 have poor diagnostic value for AZS detection

ROC curve analysis of two differently expressed genes (SEMG1 and CRISP2), alone or combined is displayed in figure 1. SEMG1 and CRISP2 expression, alone or combined, showed a poor sensitivity and specificity for identification of AZS phenotype between case and control groups.

## Discussion

AZS, a cause of infertility in men, could be caused by dysfunction of energy metabolism or structural defects in the sperm-tail proteins and the sperm motility proteins. Despite the advances in etiology of male infertility, the molecular mechanisms that impair sperm motility in most cases are unclear (34). The mRNA expression analysis of four candidate genes including CRISP2, CATSPER1, SEMG1 and PATE1 in the sperm of men with AZS and control groups showed that down-regulation of CRISP2 (p=0.036) and up-regulation of SEMG1 (p= 0.03) were associated with AZS.

Zhou et al. demonstrated that miR-27b and miR-27a negatively regulate CRISP2 protein expression in AZS and asthenoteratozoospermia, respectively (13, 35). Accordingly, high miR-27b and miR-27a expression or low CRISP2 protein expression was significantly associated with low sperm motility, abnormal morphology, and infertility in asthenoteratozoospermic men (13, 35, 36). Consistent with these studies, CRISP2 expression was downregulated in AZS samples, but this deregulation was found in mRNA. Similar to our finding, Jing et al. reported downregulation of CRISP2 mRNA (37) and protein (38) or both (39, 40) in AZS. CRISP2 protein that has been localized in the acrosome and sperm tail and involved in sperm-egg fusion, is a candidate gene in men infertility; however, analysis of CRISP2 variations in asthenozoo-and/or teratozoospermia failed to find a significant association (41). It is exciting that Agarwal et al. found CRISP2 is uniquely expressed in the spermatozoa of infertile men with unilateral varicocele and it is absent in fertile men (42). These findings suggest the involvement of CRISP2 in the infertility may be the result of impairing sperm motility and development of varicocele. Varicocele is a risk factor for sperm motility and it can significantly affect it although the pathophysiologic mechanisms are not yet completely known.

Consistent to our finding, previous studies in AZS samples have shown SEMG1 levels increase and remain bound to spermatozoa (15, 43, 44), and their mRNA is overexpressed in spermatozoa (15). Legare et al. found over-expression of SEMG1 in infertile men and men who failed to fertilize eggs during IVF procedures (45). The high expression of SEMG1 in AZS patients could cause the accumulation of Sg1, and this might increase the concentration of Sg1 and decrease the motility of sperm in AZS men (15). The Sg1, the predominant secreted protein in semen, is contributed in the formation of a gel matrix which encases the ejaculated spermatozoa. After ejaculation, Sg1 is broken down into smaller peptides by PSA. This process liquefies the semen coagulum and allows the sperm to be more motile (15, 46). Interestingly, some SEMG1 variations (Such as p.Tyr315His and p.Gly400Asp) are most likely affecting molecular interactions or protein activity and possibly leading to hyperviscosity and AZS (47).

In contrast to previous reports, no association was found between the differential expression of CATSPER1 and PATE1 with the AZS which might be related to sample size. CATSPER1 protein is a calcium channel which particularly acts in the plasma membrane of the sperm tail (27). Low, lack of or mislocalized expression of CATSPER1 protein in spermatozoa may be involved in developing asthenozoospermic phenotype and low hyperactivated motility (48–50). This low or absent expression of CATSPER1 was seen in mRNA level (49, 50). Also, some polymorphisms and mutations of CATSPER1 are associated with AZS (49, 51, 52). PATE1 protein in sperm seems to mediate sperm-egg interactions. According to a previous report, a defect in sperm PATE1 protein was revealed in both aged and young AZS men. The antibody of PATE1 blocking can decrease the motility of human sperm and zona-free hamster oocyte penetration (14). Recently, it was shown that PATE1 variant (A1423G) was possibly one of the genetic risk factors for idiopathic AZS (53).

## Conclusion

In conclusion, although our samples were limited, our finding suggested that down-and up-regulation of the CRISP2 and SEMG1 respectively was associated with the idiopathic AZS infertile men.
